# Expression of Mesenchymal Stem Cells-Related Genes and Plasticity of Aspirated Follicular Cells Obtained from Infertile Women

**DOI:** 10.1155/2014/508216

**Published:** 2014-03-03

**Authors:** Edo Dzafic, Martin Stimpfel, Srdjan Novakovic, Petra Cerkovnik, Irma Virant-Klun

**Affiliations:** ^1^Department of Obstetrics and Gynaecology, University Medical Centre Ljubljana, Šlajmerjeva 3, 1000 Ljubljana, Slovenia; ^2^Department of Molecular Diagnostics, Institute of Oncology Ljubljana, Zaloška 2, 1000 Ljubljana, Slovenia

## Abstract

After removal of oocytes for *in vitro* fertilization, follicular aspirates which are rich in somatic follicular cells are discarded in daily medical practice. However, there is some evidence that less differentiated cells with stem cell characteristics are present among aspirated follicular cells (AFCs). The aim of this study was to culture AFCs *in vitro* and to analyze their gene expression profile. Using the RT^2^ Profiler PCR array, we investigated the expression profile of 84 genes related to stemness, mesenchymal stem cells (MCSs), and cell differentiation in AFCs enriched by hypoosmotic protocol from follicular aspirates of infertile women involved in assisted reproduction programme in comparison with bone marrow-derived mesenchymal stem cells (BM-MSCs) and fibroblasts. Altogether the expression of 57 genes was detected in AFCs: 16 genes (*OCT4*, *CD49f*, *CD106*, *CD146*, *CD45*, *CD54*, *IL10*, *IL1B*, *TNF*, *VEGF*, *VWF*, *HDAC1*, *MITF*, *RUNX2*, *PPARG*, and *PCAF*) were upregulated and 20 genes (*FGF2*, *CASP3*, *CD105*, *CD13*, *CD340*, *CD73*, *CD90*, *KDR*, *PDGFRB*, *BDNF*, *COL1A1*, *IL6*, *MMP2*, *NES*, *NUDT6*, *BMP6*, *SMURF2*, *BMP4*, *GDF5*, and *JAG1*) were downregulated in AFCs when compared with BM-MSCs. The genes which were upregulated in AFCs were mostly related to MSCs and connected with ovarian function, and differed from those in fibroblasts. The cultured AFCs with predominating granulosa cells were successfully *in vitro* differentiated into adipogenic-, osteogenic-, and pancreatic-like cells. The upregulation of some MSC-specific genes and *in vitro* differentiation into other types of cells indicated a subpopulation of AFCs with specific stemness, which was not similar to those of BM-MSCs or fibroblasts.

## 1. Introduction

In infertile women, oocytes are retrieved by ultrasound-guided transvaginal follicular aspiration in the assisted reproduction programme. After removal of oocytes for *in vitro* fertilization, follicular aspirates which are rich in somatic follicular cells are discarded in daily medical practice. Each follicular aspirate consists of numerous types of somatic cells along with follicular fluid [1]. The main types of aspirated follicular cells (AFCs) are represented by granulosa cells (GCs) and theca cells (TCs). The main role of GCs is to support the oocyte by providing some nutrients that are essential for oocyte growth and development and to accumulate the metabolites secreted by the oocyte. On the other hand, TCs produce androgens which are converted to estradiol by GCs [2]. Nevertheless, the follicular aspirate is also composed of other types of cells such as red and white blood cells thus reflecting good vascularization and some resident immune cells in ovarian follicles. Moreover, also some vaginal and ovarian surface epithelial cells can be present among AFCs since these tissues are penetrated during transvaginal follicular aspiration [3, 4].

Follicular aspirates are discarded in daily medical practice but could be an important source for potential research, diagnostics (e.g., immunoassays), and cell therapy in the future, since it has already been evidenced that subpopulations of AFCs can express some stem cell characteristics [5]. Especially, GCs represent a very interesting subpopulation of AFCs as demonstrated by several studies and recently reviewed by our group [6]. GCs originate from ovarian surface epithelium and form the major part of the growing follicle, possess a remarkable proliferation activity, and represent a predominant type of AFCs [7]. Studies evidenced expression of the stemness-related marker *OCT4* and multiple mesenchymal linage-related markers in GCs along with their differentiation into other types of cells [8], especially spontaneous differentiation into osteogenic-like cells [9]. Moreover, the possible contribution of less differentiated GCs in development of ovarian cancers has been suggested [10]. Along with GCs, it has also been shown that subpopulation of TCs contains putative stem cells [11].

It is of great scientific interest to isolate, proliferate, and research the less differentiated/progenitor cells among AFCs for potential medical use in the future. However, there have been no studies until now which would analyze the broader gene expression profile of AFCs and elucidate the potential relation of AFCs to mesenchymal stem cells (MSCs), the most common cells tested in the regeneration of impaired ovarian function in the animal models [12, 13].

The aim of this study was therefore to analyse the expression of eighty-four different genes related to stemness (pluripotency), MSCs, and cell differentiation in cultured AFCs from follicular aspirates of infertile women included in the assisted reproduction programme in comparison with bone marrow-derived MSCs (BM-MSCs) and human dermal fibroblasts (HDFs). We also tested the osteogenic, adipogenic, and pancreatic differentiation in cultured AFCs to evidence their plasticity. Our results showed that cultured AFCs expressed specific stemness related to MSCs but other than in BM-MSCs and somatic fibroblasts. Moreover, the cultured AFCs were able to differentiate into adipogenic-, osteogenic-, and pancreatic-like cells *in vitro*.

## 2. Materials and Methods

### 2.1. Collection of AFCs

This study was approved by the Slovenian Medical Ethical Committee (Ministry of Health, number 196/10/07). After written informed consents, follicular aspirates were collected by transvaginal ultrasound-guided aspiration from twelve infertile patients treated with controlled ovarian hyperstimulation for assisted reproduction. Patients were treated with various exogenous gonadotropins as described previously [14]. After removal of the cumulus oophorus-oocyte-complexes, the AFCs were enriched from the follicular aspirates using hypoosmotic technique as described by Lobb and Younglai [15], mainly to remove red blood cells. Briefly, the freshly collected follicular aspirates from each patient were pooled in conical bottomed 50 mL polypropylene centrifuge tubes and centrifuged at 1400 rpm for 6 min. The supernatant was aspirated and the remaining cell slurry was pipetted into a 15 mL conical bottomed polystyrene centrifuge tube. To the cell slurry 9.0 mL of sterile distilled water was added and the tube was capped and mixed. After 60 s, 1.0 mL of 10x concentrated phosphate buffer saline (PBS; pH 7.4) was added and the tube was capped and mixed. The tubes were then centrifuged at 800 rpm for 3 min; the supernatant was discarded; the cell pellet was resuspended in 0.5 mL of culture medium and transferred into a culture dish. From each patient, one AFCs culture was established.

### 2.2. Cell Cultures

Cells were cultured in gelatin-coated 4-well culture dish (15 mm well diameter) at concentration of 1 × 10^5^ cells per well. For the culture medium, DMEM/F12 (Sigma-Aldrich) with 20% follicular fluid serum (FF) retrieved from the *in vitro* fertilization programme was used. FF was prepared as described previously by Stimpfel et al. [16]. The cells were cultured in a CO_2_ incubator at 37°C and 6% CO_2_ in air and daily monitored at the heat-staged inverted microscope (Nikon, Japan). When the cell culture was set up, the culture medium was replaced by a fresh medium on the next day to remove the remaining red blood cells. The cell splitting was performed when needed using 0.15% trypsin (Sigma-Aldrich). Alive AFCs were maintained in a cell culture based on two criteria: (i) cells were attached to the surface of culture dish and (ii) cells proliferated. The cells were cultured up to 2 months.

### 2.3. Gene Expression Analysis

Human Mesenchymal Stem Cell RT² Profiler PCR Array (PAHS-082, SABiosciences, Qiagen) was used to evaluate the expression of 84 specific genes related to stemness (pluripotency), MSCs, and cell differentiation—osteogenesis, adipogenesis, chondrogenesis, myogenesis, and tenogenesis (see Supplementary Table  1 available online at http://dx.doi.org/10.1155/2014/508216). After 5 days of culturing, three AFCs cultures from three different patients who aged 36 years (uterine abnormality), 36 years (no indication of infertility/male infertility), and 38 years (tubal factor of infertility) were pooled together and analysed along with control samples. As a positive control, a commercially available cell line of bone marrow-derived mesenchymal stem cells (BM-MSCs) was used (Chemicon, Millipore, cat. number SCC034). These cells were cultured in a mesenchymal stem cell expansion medium provided by the same producer (cat. number SCM015). As a negative control, adult human dermal fibroblasts (HDFs) were used (Cascade Biologics, Invitrogen, cat. number C-013-5C), which were cultured in DMEM/F12 (Sigma-Aldrich) with 10% FBS (Gibco, Invitrogen).

The total RNA was isolated from 10^5^ to 10^6^ cells using the miRNeasy Mini kit (Qiagen) according to the manufacturer's instructions. cDNA was synthesized from 500 ng of the total RNA using the RT^2^ First Strand Kit (Qiagen), which includes the additional removal of genomic DNA from the RNA sample and a specific control of reverse transcription. The quality of isolated RNA was also evaluated using RT^2^ RNA QC PCR Arrays (Qiagen) according to the manufacturer's instructions. This test includes various measures allowing to control the presence of reverse transcription and PCR inhibitors, contamination with genomic DNA, and contamination with DNA during the procedure.

After all control tests, the samples were analysed using the RT² Profiler PCR Array. Altogether, 84 different genes were simultaneously amplified in the sample. A melting curve analysis was performed to verify that the product consisted of a single amplicon. PCR arrays were performed in 384-well plates on a LightCycler 480 instrument (Roche Applied Science). Briefly, the reaction mix was prepared from 2x SABiosciences RT^2^ qPCR Master Mix and 102 *μ*L of sample cDNA. 10 *μ*L of this mixture was added into each well of the PCR Array. The data were analysed via Roche LightCycler 480 software and the *C*_*t*_ values were extracted for each gene. The thresholds and baselines were set according to the manufacturer's instructions (SABiosciences, Qiagen). The data were analysed using software supplied by Qiagen (http://www.sabiosciences.com/pcr/arrayanalysis.php). The fold change in gene expression (compared to positive control BM-MSCs) was calculated using the ΔΔ*C*_*t*_ method. A more than threefold change in gene expression (compared to positive control BM-MSCs) was considered as the up- or downregulation of a specific gene expression.

### 2.4. Alkaline Phosphatase Activity Staining

An alkaline phosphatase detection kit (Millipore) was used for staining of alkaline phosphatase (AP) activity. Briefly, the AFCs were fixed in 4% paraformaldehyde (PFA) for 1 min, washed with PBS, and incubated for 15 min in a working solution of reagents, which consisted of Fast Red Violet, Naphthol AS-BI phosphate solution and water in a 2 : 1 : 1 ratio. The culture was observed under an inverted microscope (Hoffman illumination) to confirm AP activity. The cells or cell clusters expressing AP activity were stained from pink to violet.

### 2.5. Differentiation of AFCs into Osteogenic-, Adipogenic-, and Pancreatic-Like Cells

Osteogenic differentiation was induced using the well-known osteogenic differentiation medium [17]. It consisted of DMEM low glucose, L-glutamine, FBS, dexamethasone (Sigma), L-ascorbic acid 2-phosphate (Sigma), *β*-Glycerophosphate (Sigma), and penicillin/streptomycin. To confirm successful differentiation, the cell culture was stained using the von Kossa protocol after 12–14 days of differentiation. The cells were fixed in a 4% PFA, incubated in 2% silver nitrate in the dark for 10 minutes, washed with distilled water, and exposed to UV-light for 25 minutes. After washing, the cells were observed under an inverted microscope to detect the calcium deposits, which were stained black.

To induce adipogenic differentiation, an induction medium was used as previously described [16]. The cells were cultured in a medium consisting of hESC medium (DMEM/F12, 20% KnockOut Serum Replacement (Gibco), 1 mM L-glutamine (PAA), 1% nonessential amino acids (PAA), 0.1 mM 2-mercaptoethanol (Invitrogen), 13 mM HEPES, 8 ng/mL human basic fibroblast growth factor (bFGF, Sigma-Aldrich), and 1% penicillin/streptomycin) and 20% FF. The differentiation medium was changed every 3-4 days. After 2 weeks, the cells were fixed in a 4% PFA for 20 minutes and incubated for 10 minutes in an Oil Red O working solution. After thorough washes, the cells were observed under an inverted microscope for presence of lipid droplets, which were stained red.

To induce pancreatic differentiation, the cells were cultured according to the protocol of Chandra et al. [18] which was slightly modified. Briefly, the cells were cultured for two days in SFM medium (serum free medium; DMEM/F12, 1% ITS, 1% BSA) supplemented with 4 nM activin A, 50 *μ*M 2-mercaptoethanol, and 2 ng/mL bFGF. On the third day, the medium was changed to SFM supplemented with 0.3 mM taurine and on the fifth day to SFM supplemented with 3 mM taurine, 1 mM nicotinamide, and 1% nonessential amino acids. After 10–14 days, the cells were analysed by using dithizone staining. Briefly, the stock solution of dithizone was prepared by dissolving 10 mg of dithizone in 1 mL of dimethyl sulfoxide (DMSO). Then, 10 *μ*L of stock solution was added to 1 mL of DMEM/F12 and filtered through a 0.4 *μ*m filter, and cells were incubated in this working solution for 15 min at 37°C. After incubation, the cells were washed 4 times with PBS and observed under an inverted microscope. Positively stained cells were coloured red.

## 3. Results

### 3.1. Expression of MSCs-Related Genes in AFCs and Fibroblasts in Comparison to BM-MSCs

**Figure 1:**
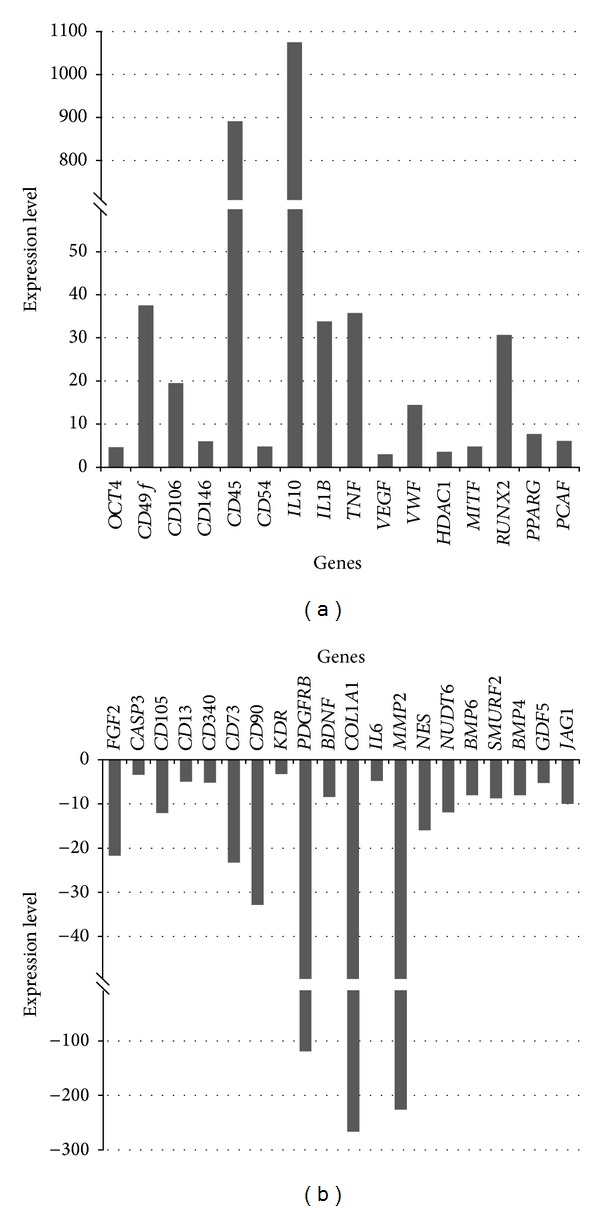
Expression levels of upregulated (a) and downregulated (b) genes in aspirated follicular cells obtained from follicular aspirates when compared with bone marrow-derived mesenchymal stem cells (positive control).

Expression of 57 genes was detected in AFCs when compared with BM-MSCs (positive control) (Table 1). Sixteen genes were upregulated in AFCs, among which MSC-associated genes *IL10* and *CD45* were two of the most upregulated genes with fold change of almost 1100 and 900, respectively. Fold change between 30 and 40 was detected for MSC-specific or associated genes *CD49f*, *TNF*, *IL1B*, and adipogenesis- and osteogenesis-related *RUNX2*. Two highly upregulated genes were also MSC-specific or associated genes *CD106* and *VWF* with fold change of around 20 and 15, respectively. All other genes (*OCT4*, *CD146*, *CD54*, *VEGF*, *HDAC1*, *MITF*, *PPARG*, and *PCAF*) showed fold change between 3 and 10 (Figure 1(a)). Twenty genes were downregulated in AFCs when compared with BM-MSCs, among which MSC-specific or associated genes *COL1A1*, *MMP2*, and *PDGFRB* were the most downregulated genes with fold changes −266 (*COL1A1*), −225 (*MMP2*), and −119 (*PDGFRB*). Highly downregulated genes were also *FGF2*, *CD73*, *CD90*, *NUDT6*, *NES*, and *CD105,* with fold changes between −33 and −12, respectively. All other genes (*GDF5*, *CASP3*, *CD13*, *CD340*, *KDR*, *BDNF*, *IL6*, *BMP6*, *SMURF2*, *BMP4*, and *JAG1*) showed fold change between –3 and –10 (Figure 1(b)). There were 27 genes which were not detected in AFCs; about one-third of them was stemness or MSCs-specific genes; one-third was genes associated with MSCs, and one-third was osteogenesis- or chondrogenesis-related genes. All these data showed that cultured AFCs expressed several genes specific or associated with MSCs, but the expression pattern was different than in BM-MSCs. Similar to BM-MSCs, AFCs did not express the key genes related to stemness or pluripotency (*SOX2*, *REX1*, *TERT*, *WNT3A*, and *INS*) or expressed them at very low level (*OCT4* and *LIF*).

**Figure 2:**
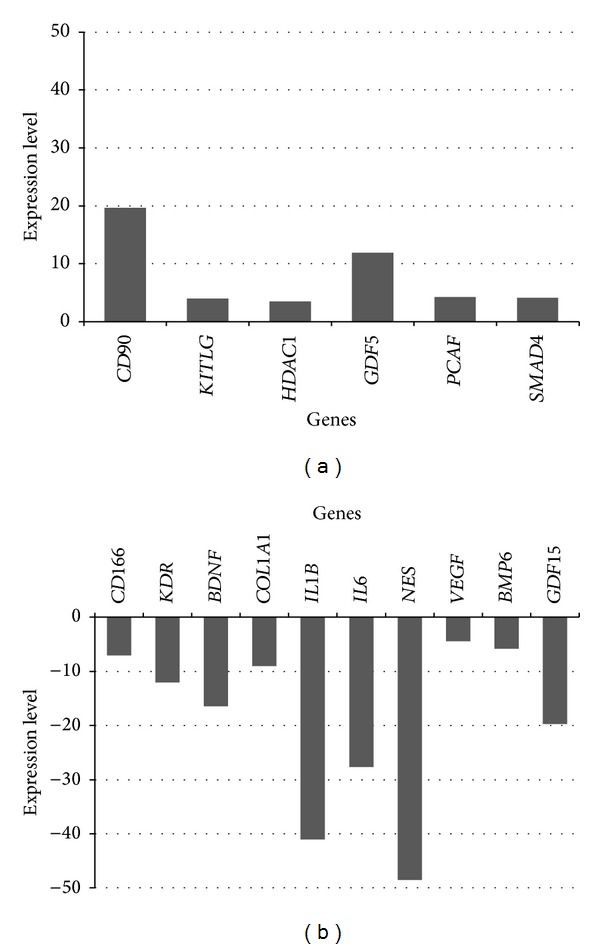
Expression levels of upregulated (a) and downregulated (b) genes in adult human dermal fibroblasts (negative control) when compared with bone marrow-derived mesenchymal stem cells (positive control).

In AFCs, there was a higher number of upregulated genes than in HDFs (negative control) in comparison with BM-MSCs. In AFCs, other set of MSC-specific or associated genes (*CD49f*, *CD106*, *CD146*, *CD45*, *CD54*, *IL10*, *IL1B*, *TNF*, *VEGF*, and* VWF*) were prominently upregulated than in HDFs (*CD90* and *KITLG*). In HDFs, the expression of lower number of genes was detected than in AFCs. The expression of 50 genes was detected in HDFs when compared with BM-MSCs (Table 1). A lower number −6 genes were upregulated in HDFs, among which MSC-specific *CD90* was the most upregulated gene with fold change of around 20. All other upregulated genes (*KITLG*—associated with MSCs, *HDAC1*—osteogenesis, *PCAF*—chondrogenesis, and *SMAD4*—tenogenesis) had fold change of around 4, with exception of chondrogenesis-related *GDF5*, which had fold change of around 10 (Figure 2(a)). Ten genes were downregulated in HDFs when compared with BM-MSCs, among which MSC-specific *NES*, *IL1B,* and *IL6* were the most downregulated genes with fold change of around −30 (*IL6*), of around −40 (*IL1B)*, and of around −50 (*NES*). Fold change between −10 and −20 was detected for *GDF15*, *BDNF*, and *KDR* genes. All other genes (*CD166*, *COL1A1*, *VEGF*, and *BMP6*) showed fold change between −10 and −3 (Figure 2(b)).

### 3.2. Culturing of AFCs and Differentiation in Other Cell Types

**Figure 3:**
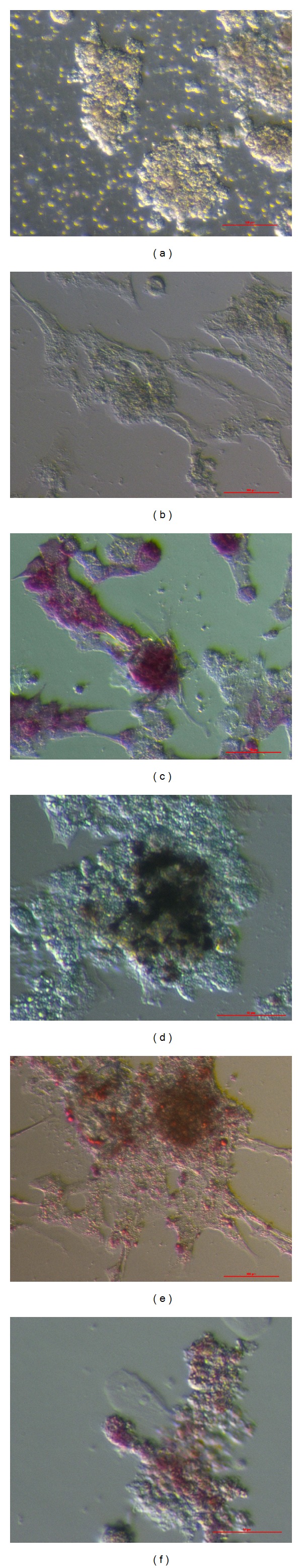
Epithelial-like phenotype of aspirated follicular cells (AFCs) in culture dish immediately after enrichment with hypoosmotic method (a). Fibroblast-like phenotype of AFCs in culture dish 48 hours after isolation (b). AFCs positive for alkaline phosphatase activity (pink-violet) (c). Differentiation of AFCs into osteogenic-like cells, von Kossa-positive staining (d). Differentiation of AFCs into adipogenic-like cells, accumulation of lipid droplets (dark red) (e). Differentiation of AFCs into pancreatic-like cells, dithizone-positive (bright red-pink) cell cluster (f). Scale bar: 100 *μ*m (a, b, c, e) and 50 *μ*m (d, f).

Immediately after transferring enriched AFCs from follicular aspirates into culture dish, we observed clusters of AFCs with approximately 100 *μ*m in diameter and also single AFCs with numerous surrounding red blood cells (Figure 3(a)) which were not removed with hypoosmotic protocol. After AFCs were attached to a culture dish surface, red blood cells were removed upon washing with PBS and first change of the culture medium (on the second day). AFCs exhibited fibroblast-like phenotype (Figure 3(b)), although epithelial-like AFCs were also observed in minority. After 48 hours, AFCs also started migrating from packed clusters. We were able to maintain AFCs alive for 2 months; however, viability (attachment to the surface and cell proliferation) of AFCs decreased with every passage, but it was unique case with every patient.

Cultured AFCs were highly positive for AP, and around 60% AFCs showed strongly pink-violet staining (Figure 3(c)) throughout the culturing. When AFCs were exposed to media for osteogenic differentiation, cell morphology was slightly changed; they shrunk, and around 10% of AFCs stained positively for mineralization (Figure 3(d)). Additionally, when AFCs were exposed to media for adipogenic differentiation, accumulation of lipid droplets was observed throughout the cell culture (Figure 3(e)). AFCs were also exposed to media for pancreatic differentiation. Cell morphology was changed forming clusters of islet-like structures and around 5% of cells positively stained on dithizone (Figure 3(f)).

## 4. Discussion

In this study, AFCs obtained from follicular aspirates of infertile women included into the *in vitro* fertilization programme were successfully cultured and their stemness was confirmed. The gene expression profile and *in vitro* differentiation of cultured AFCs into other cell types confirmed the relation of AFCs to MSCs, but their stemness was specific and it differed from BM-MSCs and fibroblasts.

The *in vitro* culturing and research of molecular and cellular characteristics of AFCs and their subpopulations such as GCs or TCs are still difficult since there is no ultimate protocol for their purification from follicular aspirates. Subpopulations of AFCs can be isolated by flow cytometry based on the expression of specific cell marker, for example, follicle-stimulating hormone receptor (FSHR) for isolation of GCs [8]; however, this approach can lead to a loss of less differentiated/progenitor GCs which do not express FSHR yet. In this study, we used the hypoosmotic purification protocol described by Lobb and Younglai [15] to enrich AFCs because it is quite simple and can be quickly done, removes most of red blood cells from the sample, and yields more AFCs in comparison with multistep protocols. The follicular aspirates also contain a proportion of white blood cells which represent approximately 15% of all cells [19] and are unavoidable contaminant. On the other hand, these “contaminating” cells could play an important role in maintaining a more physiological ovarian stem cell niche [20].

In this study, we successfully established a long-term culture of AFCs. In previous studies, the apoptosis represented a major problem in AFCs culturing and research. However, we found for the first time that the addition of follicular fluid serum to the culture medium enables a long-term survival of AFCs *in vitro*. Because the potential use of AFCs is related to their culture and proliferation *in vitro*, we were interested in gene expression analysis of cultured AFCs more than freshly isolated. However, *in vitro* culturing can significantly affect the gene expression of cells as previously shown in human stromal cells [21]. Even more, for some AFCs like GCs, it has been demonstrated that they can undergo dedifferentiation *in vitro* and downregulation of GCs-specific genes may occur after 96 hours of culturing [9].

Our data showed that all three groups of analyzed cells expressed a proportion of MSC-specific or associated genes thus reflected the same—mesodermal—origin of cells. In spite of that, the gene expression profile of AFCs, BM-MSCs, and HDFs was different and indicated three distinct groups of cells. There were eight genes which were expressed in both the AFCs and BM-MSCs, but were not expressed in HDFs; these genes were related to stemness (*LIF*) and were MSCs-specific (*CD106 *and* CD146*), associated with MSCs (*IL10*, *CD45*, *TNF*, and *VWF*) or chondrogenesis related (*SOX9*). In AFCs, several MSCs-specific or associated genes were upregulated. The AFCs were not only characterized by a very high expression of genes *IL10* and *CD45 *that may reflect their association with MSCs, but also to a lower extent the contamination with blood cells. The gene* IL10* is known to be related to immunoregulation (inflammation), while the gene *CD45* encodes the protein belonging to the tyrosine phosphatase (PTP) family; the PTPs are known to be signaling molecules that regulate a variety of cellular processes including cell growth, differentiation, mitosis, and oncogenic transformation according to the GeneCard database.

The results of this study show that AFCs expressed several genes typical for somatic ovarian cells, especially GCs. In addition, the morphology of AFCs clusters resembled the GCs; therefore, it is not excluded that GCs represented majority of cells in our cell cultures. The expression of gene *VEGF*, vascular endothelial growth factor, was previously demonstrated in GCs and was shown to be very important factor in controlling angiogenesis during development of *corpus luteum* [22]. In addition, *CD146*, melanoma cell adhesion molecule, was shown to be expressed on human luteinizing GCs [23]. *CD49f*, also known as integrin alpha-6, has been demonstrated to be expressed on the surface of human GCs and represents a differentiation marker of GCs [24]; it was found to be more distinctive for GCs from the inner layers of follicle [25]. The gene *PPAPRG*, peroxisome proliferator-activated receptor gamma, encodes a nuclear hormone receptor which is related to steroid hormone action [26]. The activity of GCs is strongly influenced by follicle-stimulating hormone and luteinizing hormone [27]. The gene *HDAC1*, histone deacetylase 1, is one of the important regulators of human luteinizing hormone receptor gene transcription [28]. In AFCs, also some genes related to osteogenesis and adipogenesis were upregulated; *MITF* has been connected with osteogenesis [29], along with *RUNX2* [30]. In addition, *PCAF* was recently shown to acetylate *RUNX2* which leads to transcriptional activity and thus promotes osteoblast differentiation [31]. In AFCs, there was a higher number of upregulated genes related to MSCs than in HDFs in comparison with BM-MSCs and other set of MSC-specific or associated genes was prominently upregulated than in HDFs. In addition, the genes upregulated in HDFs were more related to cell differentiation (osteogenesis, chondrogenesis, tenogenesis) than to stemness thus indicating that HDFs were more differentiated cells than cultured AFCs.

The AFCs were not pluripotent stem cells, because they did not express genes related to pluripotency such as *REX1*, *SOX2*, *TERT*, and *WNT3A*. In spite of that, they expressed two pluripotency-related genes: *OCT4* and *LIF* to a lower extent. The expression of *OCT4* in AFCs probably reflects the presence of GCs as previously confirmed by other studies [8, 32, 33]. However, *OCT4* was also expressed in both BM-MSCs and HDFs to the same extent; therefore, the nonspecificity of primer for *OCT4A*, related to pluripotent stem cells [34], is not excluded. It needs to be exposed that the *LIF *gene, an important marker of stemness [35], was detected to the same extent in AFCs and BM-MSCs, but was not detected in HDF; this might reflect a lower stemness of HDFs.

A subpopulation of AFCs expressed a degree of plasticity, because we were able to successfully differentiate them into osteogenic, adipocyte and pancreatic-like cells. AFCs seem to be especially in favour of osteogenesis thus reflecting the presence of GCs, as evidenced by other studies [9, 36]. In our experiments, AFCs strongly expressed the gene *RUNX2* which is involved in osteogenesis [37] and GCs luteinization [38], differentiated into osteogenic-like cells confirmed by Von Kossa staining and stained positively for alkaline phosphatase activity which is considered as an early marker of osteogenesis [39]. Moreover, AFCs were successfully differentiated into adipose and pancreatic-like cells in this study. To our knowledge differentiation of AFCs into adipocyte and pancreatic-like cells has not been reported until now; therefore, our work additionally supports the idea about the stemness and plasticity of human AFCs.

## 5. Conclusion

In conclusion, the results of our study showed that AFCs enriched from follicular aspirates of infertile women using hypoosmotic protocol and cultured *in vitro *expressed 57 from 84 analyzed genes related to stemness, MSCs, and cell differentiation. Numerous upregulated genes were specific for MSCs or were associated with them. The expression of these genes confirmed the stemness of AFCs in our cultures; however, the gene expression profile differed from that of BM-MSCs. The gene expression profile of AFCs also differed from that of HDFs which were found to be more differentiated cells. In AFCs, also several expressed genes were related to the ovary and its function. The AFCs expressed a degree of plasticity and were successfully differentiated into other types of cells which are otherwise not present in the ovary.

## Supplementary Material

Supplementary Table 1: Functional grouped list of genes used for gene expression analysis in aspirated follicular cells obtained from follicular aspirates (MSCs = mesenchymal stem cells).

## Figures and Tables

**Table 1:** Expression levels of 84 genes in adult human dermal fibroblasts and aspirated follicular cells in comparison with bone marrow-derived mesenchymal stem cells, respectively.

Gene name	Expression level		REFSEQ (mRNA)
	Fibroblasts	Aspirated follicular cells	
*ABCB1 *	—	—	NM_000927
*ANXA5 *	2.17	−1.50	NM_001154
*BDNF *	−16.45	−8.40	NM_001143805
*BGLAP *	−1.79	−1.49	NM_199173
*BMP2 *	—	—	NM_001200
*BMP4 *	2.01	−8.06	NM_001202
*BMP6 *	−5.82	−9.32	NM_001718
*BMP7 *	—	—	NM_001719
*CASP3 *	−1.44	−3.36	NM_004346
*CD105 *	−2.64	−12.04	NM_000118
*CD106 *	—	19.56	NM_001078
*CD11c *	—	—	NM_000887
*CD13 *	1.61	−4.93	NM_001150
*CD133 *	—	—	NM_001139319
*CD146 *	—	5.98	NM_006500
*CD15 *	—	—	NM_002033
*CD166 *	−7.06	1.36	NM_001243280
*CD271 *	—	—	NM_002507
*CD29 *	1.77	1.17	NM_002211
*CD340 *	−1.65	−5.17	NM_001005862
*CD349 *	—	—	NM_003508
*CD44 *	1.51	−2.55	NM_000610
*CD45 *	—	891.44	NM_001267798
*CD49f *	1.01	37.53	NM_000210
*CD51 *	−1.55	−1.71	NM_001144999
*CD54 *	1.15	4.79	NM_000201
*CD73 *	−1.03	−23.26	NM_001204813
*CD90 *	19.70	−32.90	NM_006288
*COL1A1 *	−9.06	−266.87	NM_000088
*CSF2 *	—	—	NM_000758
*CSF3 *	—	—	NM_000759
*CTNNB1 *	2.35	1.84	NM_001098209
*EGF *	—	—	NM_001178130
*FGF10 *	—	—	NM_004465
*FGF2 *	−1.45	−21.71	NM_002006
*FUT1 *	—	—	NM_000148
*GDF15 *	−19.70	−1.89	NM_004864
*GDF5 *	11.88	−5.28	NM_000557
*GDF6 *	—	—	NM_001001557
*GDF7 *	—	—	NM_182828
*GTF3A *	−2.00	1.82	NM_002097
*HAT1 *	1.23	1.75	NM_001033085
*HDAC1 *	3.48	3.58	NM_004964
*HGF *	—	—	NM_000601
*HNF1A *	—	—	NM_000545
*IFNG *	—	—	NM_000619
*IGF1 *	—	—	NM_000618
*IL10 *	—	1074.91	NM_000572
*IL1B *	−41.07	33.83	NM_000576
*IL6 *	−27.67	−4.76	NM_000600
*INS *	—	—	NM_000207
*JAG1 *	−1.44	−9.99	NM_000214
*KDR *	−12.04	−3.25	NM_002253
*KITLG *	3.97	−1.87	NM_000899
*LIF *	—	−1.20	NM_001257135
*MITF *	1.63	4.82	NM_000248
*MMP2 *	1.97	−225.97	NM_001127891
*NES *	−48.50	−16.00	NM_006617
*NOTCH1 *	—	—	NM_017617
*NUDT6 *	−1.49	−11.88	NM_007083
*OCT4 *	2.27	4.63	NM_001173531
*PCAF *	4.23	6.11	NM_003884
*PDGFRB *	2.30	−119.43	NM_002609
*PIGS *	−1.49	2.48	NM_033198
*PPARG *	1.80	7.67	NM_005037
*PTK2 *	1.04	−1.14	NM_001199649
*REX1 *	—	—	NM_174900
*RHOA *	−1.15	−1.52	NM_001664
*RUNX2 *	1.68	30.70	NM_001015051
*SLC17A5 *	2.25	−1.51	NM_012434
*SMAD4 *	4.11	2.06	NM_005359
*SMURF1 *	1.14	1.23	NM_001199847
*SMURF2 *	−2.51	−8.75	NM_022739
*SOX2 *	—	—	NM_003106
*SOX9 *	—	1.72	NM_000346
*TBX2 *	—	—	NM_005994
*TERT *	—	—	NM_001193376
*TGFB1 *	−1.54	2.03	NM_000660
*TGFB3 *	1.01	1.28	NM_003239
*TNF *	—	35.75	NM_000594
*VEGF *	−4.44	3.10	NM_001025366
*VIM *	−1.53	−1.87	NM_003380
*VWF *	—	14.42	NM_000552
*WNT3A *	—	—	NM_033131

—: expression of the gene was not detected.
