# Supplementary material for: Expression of Mesenchymal Stem Cells-Related Genes and Plasticity of Aspirated Follicular Cells Obtained from Infertile Women
Source: Biomed Res Int. 2014 Mar 3;2014:508216. doi: 10.1155/2014/508216 (PMC3958784; doi:10.1155/2014/508216)
Supplement: Supplementary file 1 — Supplementary Table 1: Functional grouped list of genes used for gene expression analysis in aspirated follicular cells obtained from follicular aspirates (MSCs = mesenchymal stem cells). [file 508216.f1.pdf]

Supplementary Data

Table 1: Functional grouped list of genes used for gene expression analysis in aspirated follicular cells obtained from follicular aspirates (MSCs = mesenchymal stem cells).

FUNCTIONAL GENE GROUPING	GENE NAMES
**Stemness (pluripotency) markers**	*FGF2, INS, LIF, OCT4, REX1, SOX2, TERT, WNT3A*
**MSCs–specific markers**	*BMP2, CASP3, CD105, CD106, CD13, CD133, CD146, CD15, CD166, CD271, CD340, CD349, CD44, CD49f, CD51, CD73, CD90, KDR, PDGFRB*
**Associated with MSCs**	*ANXA5, BDNF, BGLAP, BMP7, CD29, CD45, CD54, COL1A1, CSF2, CSF3, CTNNB1, EGF, FUT1, GTF3A, HGF, IFNG, IGF1, IL10, IL1B, IL6, KITLG, MMP2, NES, NUDT6, PIGS, SLC17A5, TGFB3, TNF, VEGF, VIM, VWF*
**Osteogenesis**	*BMP2, BMP6, FGF10, HDAC1, HNF1A, KDR, MITF, PTK2, RUNX2, SMURF1, SMURF2, TBX2*
**Adipogenesis**	*PPARG, RHOA, RUNX2*
**Chondrogenesis**	*ABCB1, BMP2, BMP4, BMP6, CD11c, GDF5, GDF6, GDF7, HAT1, PCAF, SOX9, TGFB1*
**Myogenesis**	*ACTA2, JAG1, NOTCH1*
**Tenongenesis**	*BMP2, GDF15, SMAD4, TGFB1*
